# Mathematical model and stability analysis on the transmission dynamics of skin sores

**DOI:** 10.1017/S0950268822001807

**Published:** 2022-11-18

**Authors:** Abayneh Kebede Fantaye, Masitawal Demsie Goshu, Berhanu Belay Zeleke, Adane Abebaw Gessesse, Mehari Fentahun Endalew, Zerihun Kinfe Birhanu

**Affiliations:** 1 Department of Mathematics, Debre Tabor University, Debre Tabor, Ethiopia; 2 Department of Mathematics, Hawassa University, Hawassa, Ethiopia

**Keywords:** Basic reproduction number, modelling, skin sores, stability analysis

## Abstract

In this study, a non-linear deterministic model for the transmission dynamics of skin sores (impetigo) disease is developed and analysed by the help of stability of differential equations. Some basic properties of the model including existence and positivity as well as boundedness of the solutions of the model are investigated. The disease-free and endemic equilibrium were investigated, as well as the basic reproduction number, *R*_0_, also calculated using the next-generation matrix approach. When *R*_0_ < 1, the model's stability analysis reveals that the system is asymptotically stable at disease-free critical point globally as well as locally. If *R*_0_ > 1, the system is asymptotically stable at disease-endemic equilibrium both locally and globally. The long-term behaviour of the skin sores model's steady-state solution in a population is investigated using numerical simulations of the model.

## Introduction

Skin sores (impetigo) is a highly contagious, superficial skin infection that most commonly affects children [1], which increases in prevalence in late summer [2]. *Streptococcus pyogenes* (group A *Streptococcus*) is one of the most important bacterial causes of skin and soft tissue infections worldwide [3]. Impetigo most typically affects exposed parts, such as the face and extremities. The lesions are well-localised, however they are frequently numerous and might be bullous or non-bullous [4].

*Staphylococcus aureus* and *Streptococcus pyogenes* produce this epidermal infection [5]. Although *Staphylococcus aureus* is the most common cause of impetigo in developed countries, *S. pyogenes* is still a major cause in developing countries [6]. Infections of the skin are caused primarily by group A streptococci (GAS) [7]. GAS infections have the potential to become invasive and can lead to serious post-infectious consequences of glomerulonephritis and rheumatic heart disease [8].

The age-specific prevalence of skin sores among children of up to 14 years of age is high, but remarkably consistent across the age groups studied [9]. Impetigo has increased occurrence in close contact, warm and humid environments [10]. *In vitro* investigations are used to assess the efficacy of prospective novel antimicrobial agents, with *in vivo* studies in animal models and/or people following if they are successful [11]. According to recent estimates, impetigo affects anywhere from 111 million children in impoverished nations to 140 million people worldwide at any given moment [12].

**Fig. 1.**
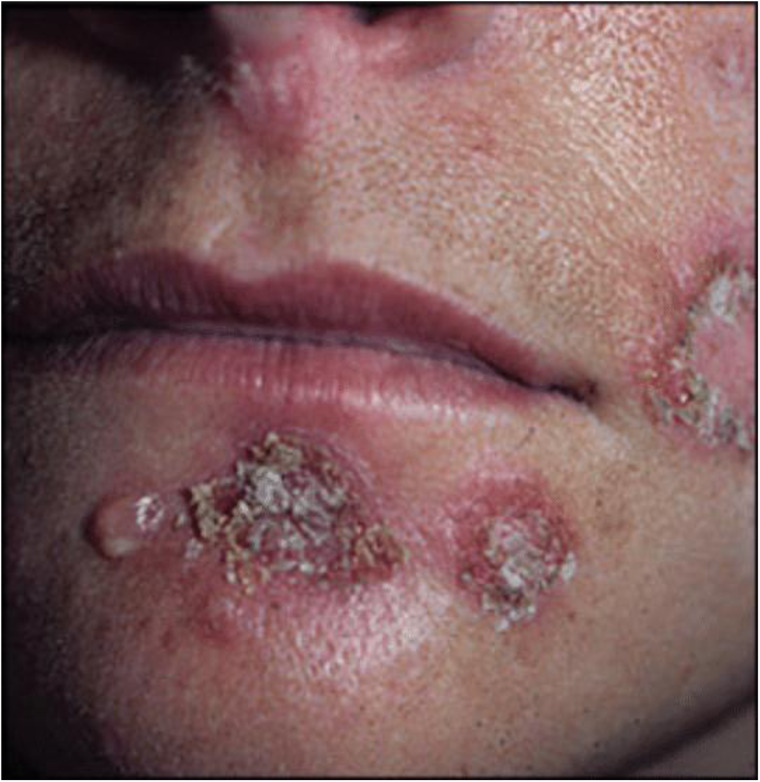
Non-bullous impetigo.

**Fig. 2.**
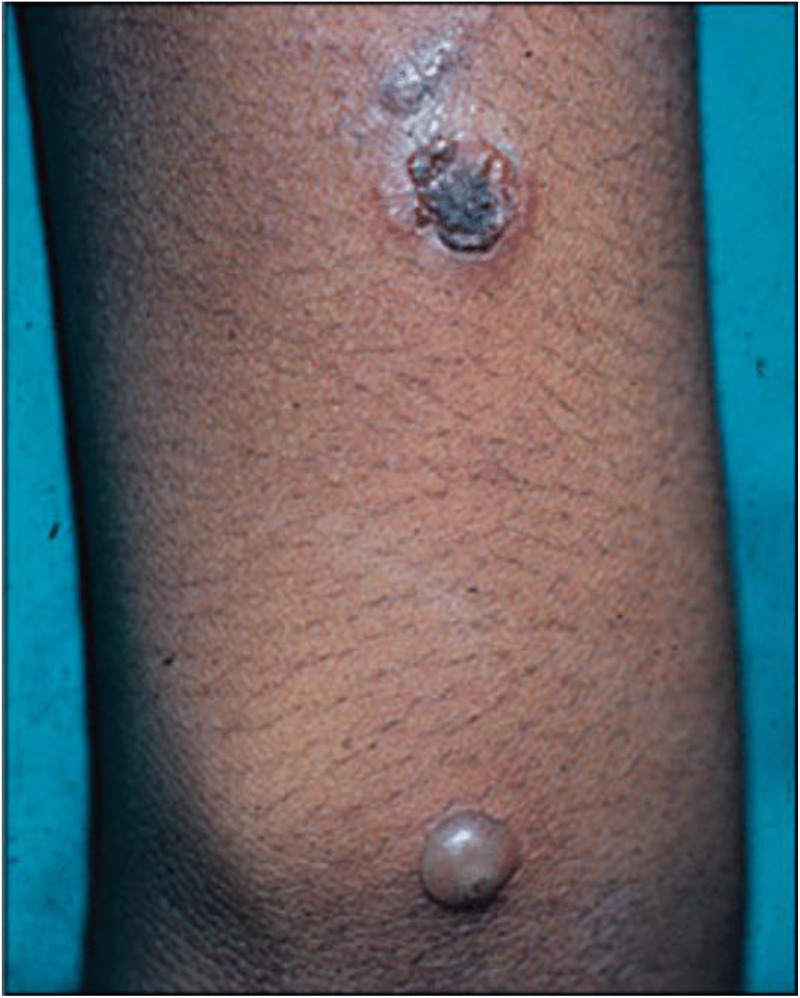
Bullous impetigo.

Impetigo is divided into non-bullous (also known as impetigo contagiosa) and bullous types. Bullous impetigo is caused by a staphylococcal toxin and does not require a host response to manifest clinical disease, whereas non-bullous impetigo is an infection-related host response [1] (Figs 1 and 2).

In recent years, mathematical modelling is becoming key techniques in the area of infectious disease propagation and control [13–18]. The studies on the mathematical analysis of human diseases and epidemic models have been conducted, which combine numerical studies with dynamic system methodologies such as stability analysis, LaSalles invariance principle, Routh–Hurwitz criterion and Lyapunov function [19].

Tanaka and Ono [20] provide an overview of how mathematical modelling can improve understanding of skin diseases, considering both their benefits and challenges. They discuss the significance of strong linkages between experimental, mathematical modelling and data analysis in order to effectively utilise the modelling technique to improve skin research in the post-genomic era.

Lydeamore *et al*. [7] constructed a stochastic representation of the susceptible-infectious-susceptible model. They have provided the first model-based estimates of skin sores infection duration (between 12 and 20 days), infection strength and baseline reproductive ratio in three different settings. By performing the estimation in a modelling framework, the interval-censored nature of the data was taken into account.

Motivated by the works of [7], for this manuscript, a non-linear deterministic model to study and analyse the transmission of skin sores is proposed. Moreover, we subdivide the total population into susceptible (*J*(*t*)), infected (*K*(*t*)) and recovered (*K*(*t*)) classes.

The manuscript is organised by four sections. In the first section, introduction to the transmission disease skin sores is presented. We construct a mathematical model for the dynamics of skin sore transmission in section ‘Model formulation’. Additionally, we go over the fundamental characteristics of the model and stability analysis of the disease-free and endemic equilibria. The topic of numerical simulation is covered in section ‘Numerical simulation’. At last, the conclusion is provided in section ‘Conclusion’.

## Model formulation

**Fig. 3.**
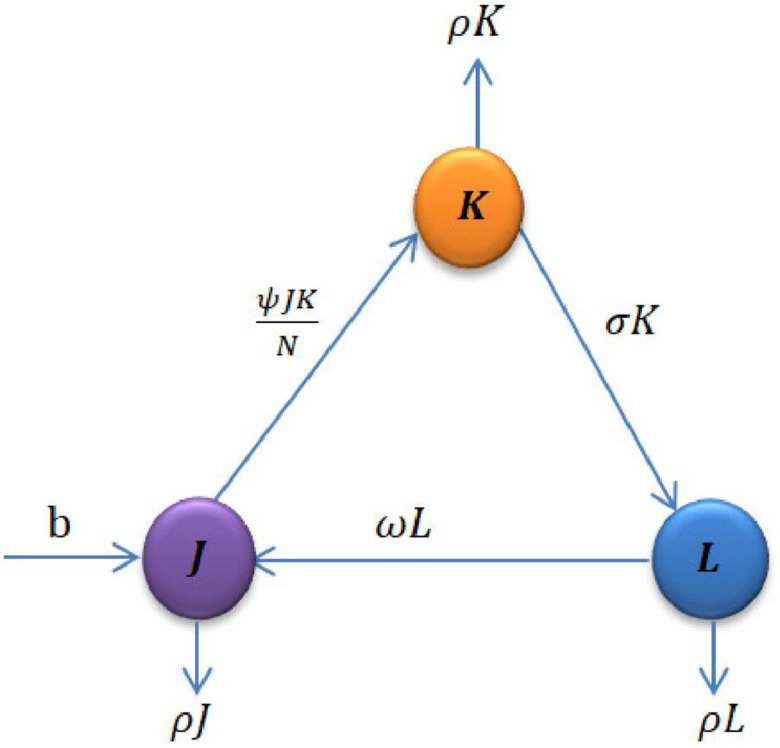
Flow chart of the model.

In this section, we develop a new mathematical model for skin sores transmission dynamics by dividing the total population *N*(*t*) into three compartments: susceptible (*J*(*t*)), those who are at risk of becoming infected by the disease; infected (*K*(*t*)), those who have the pathogen in their organism and can transmit it; and recovered (*L*(*t*)), those who have recovered from the disease. The model also assumes that there is a positive recruitment rate *b* into susceptible class (*J*(*t*)) and positive natural death rate *ρ* for all time under the study. The susceptible individuals can become infected at rate (*ψJ*/*N*) through contact with infected individuals which are depending on time, where *ψ* is effective contact rate. The remaining model parameters are likewise defined as follows: *σ* is the recovery rate and *ω* is the rate of susceptibility of recovered individuals (Fig. 3, Table 1).

**Table 1.** Parameters of the model

Parameter	Description
*b*	Recruitment rate
*ψ*	Effective contact rate
*σ*	The recovery rate
*ω*	The rate of susceptibility of recovered individuals
*ρ*	Natural death rate

Based on the assumption and flow chart of the model, we have the following system of non-linear ordinary differential equations:1
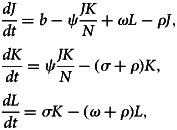
with2
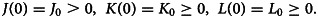


### Existence, finiteness and non-negativity of solutions

#### Existence of solution

The mathematical model of our system is well-posed. In actuality, Picard's (or Cauchy–Lipschitz) theorem states that its solution exists, is unique and is constantly dependent on the initial data. Because we are dealing with human population, we must guarantee that our solutions are positive and bounded.

#### Positivity of solution

*Theorem 1:* If 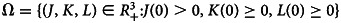, then the solution set {*J*(*t*), *K*(*t*), *L*(*t*)} of the system of equation (1) is non-negative for all *t* ≥ 0.

*Proof:* We assume that 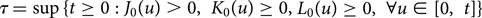. Since *J*_0_(*t*) > 0, *K*_0_(*t*) ≥ 0 and *L*_0_(*t*) ≥ 0, then *τ* ≥ 0. If *τ* < 0, then automatically *J*_0_(*t*) or *K*_0_(*t*) or *L*_0_(*t*) = 0 at *τ*. Now, use the system of equation (1)3
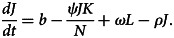


The answer to equation (3) at time *t* is thus given by applying a variant of the constant formula.4
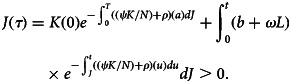


Furthermore, because of the non-negative in [0, *τ*], *J*(*τ*) > 0. Similar evidence demonstrates that *K*(*τ*) ≥ 0, and *L*(*τ*) ≥ 0, this leads to contradiction. Thus, *τ* = ∞. As a result, for *t* ≥ 0, the solutions are positive.

#### Invariant region

*Lemma 1:* Suppose that the following system's initial conditions are all positive in 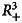.5
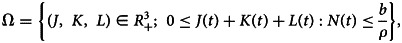
where Ω is essentially invariant.

*Proof:* Finding the derivatives of *N*(*t*) = *J*(*t*) + *K*(*t*) + *L*(*t*) by considering time as independent variable and using equation (1), we have6
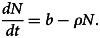


After some simplification,7
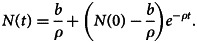


Then, 
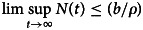
. Hence, Ω is positively invariant. Therefore, all solutions of the system given in (1) including initial condition are in Ω.

### Disease-free critical points

Disease-free critical points (DFE), *E*_0_ are solutions that reach a stable state, in which the population is free of disease. In the absence of disease, we have *K* = 0. Then, the disease-free equilibrium points (DFE), *E*_0_, are given by:8
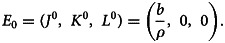


*The basic reproduction number* (*R*_0_): The basic reproduction number *R*_0_ of mathematical model of the system of equation (1) is determined using next-generation matrix technique [21]. Now, let *x* = (*K*, *L*, *J*), then the system of equation (1) can be rewritten as:9
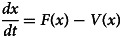
where10
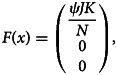
and11
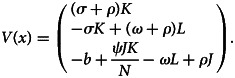


The Jacobian matrices of *F*(*x*) and *V*(*x*) at disease-free equilibrium point, *E*_0_ is given by:12
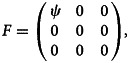
and13
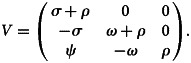


Using the method of next-generation matrix, the fundamental reproduction number, *R*_0_ is the spectral radius of *FV*^−1^ or the dominant eigenvalue of *FV*^−1^ and thus, the fundamental reproduction number *R*_0_ is given by:14
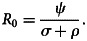


### Endemic critical point

Disease endemic critical point, *E*_1_, is said to be steady state if the disease continues in the population. If *E*_1_ = (J*, K*, L*) is disease equilibrium point, it satisfies the following algebraic equations:15
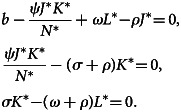


In the system (15) of the second equation, we obtain:16
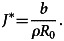


Similarly, in system (15) of the third equation, we get:17
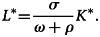


Substituting equations (16) and (17) in system (15) of the first equation, we have:18
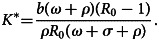


Then, after substituting equation (18) in equation (17), we obtain:19
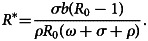


### Local stability of the disease-free equilibrium point

The linearised form of the system of equation (1) at the steady state can be used to discuss the local stability of the disease-free equilibrium.

*Theorem 3:* If *R*_0_ < 1, the equilibrium solution of the non-linear system (1) is locally asymptotically stable.

*Proof:* At the point of equilibrium without disease, the Jacobian matrix of system given in (1) is:20
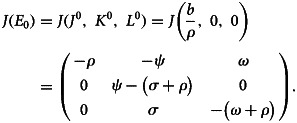


The characteristic equation of (20) at disease-free equilibrium point *E*_0_ is
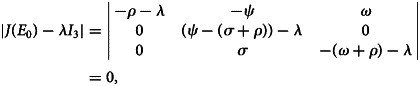
21
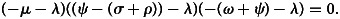


Consequently,22
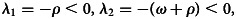
23
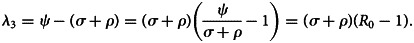


Here, *λ*_3_ = (*σ* + *ρ*)(*R*_0_ − 1) < 0 if *R*_0_ < 1 implying that all the eigenvalues are negative. Thus, disease-free equilibrium point *E*_0_ is locally asymptotically stable.

### Global stability of the disease-free equilibrium point

*Theorem 4:* Suppose that *R*_0_ < 1, then the disease-free equilibrium point *E*_0_ is globally asymptotically stable.

*Proof:* To prove the global stability of the disease-free equilibrium point *E*_0_, we need to construct the following Lyapunov function:24
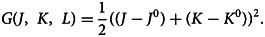


Clearly, (*G*(*J*, *K*, *L*)) ≥ 0 at disease-free equilibrium point and equal to zero at *J* = *J*^0^ and *K* = *K*^0^. Then, the derivative of equation (24) with respect to time *t* becomes:25
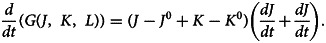


Substituting the values of (*dJ*/*dt*) and (*dK*/*dt*) from the system of equation (1), we have26
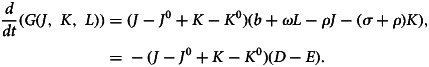


Clearly, (*d*/*dt*)(*G*(*J*, *K*, *L*)) ≤ 0 if and only if *D* > *E*, where *D* = *ρJ* + (*σ* + *ρ*)*K* and *E* = *b* + *ωL*. Furthermore, (*d*/*dt*)(*G*(*J*, *K*, *L*)) = 0 if and only if *J* = *J*^0^ and *K* = *K*^0^. Thus, by the invariance principle of LaSalle [22], the disease-free equilibrium point *E*_0_ is globally asymptotically stable.

### Local stability of the endemic equilibrium point

Here, to illustrate the local stability of the endemic disease equilibrium state, the Jacobian stability method is used.

*Theorem 5:* The system of (1) has locally asymptotically stable equilibrium solution *E*_1_, when *R*_0_ > 1.

The Jacobian matrix of the system given in (1), at the disease endemic equilibrium point, is:27
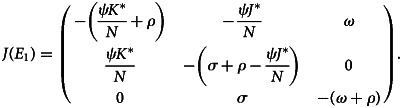


The characteristic equation of (27) at disease endemic equilibrium point *E*_1_
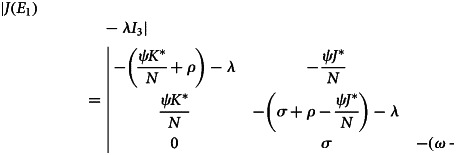


That is28
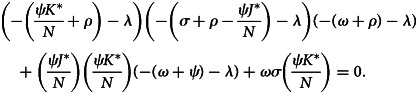


Now, equation (28) can be simplified as29
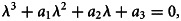
where
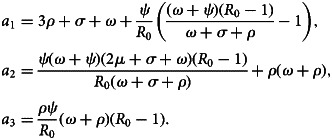


Thus, by Routh–Hurwitz criterion, the system of equation (1) is locally asymptotically stable if *a*_1_ > 0, *a*_2_ > 0 and *a*_3_ > 0. Hence, *E*_1_ is locally asymptotically stable if *R*_0_ > 1.

### Global stability of disease endemic equilibrium point

For examining the global asymptotic stability of the disease endemic equilibrium point *E*_1_, the following model is used.30
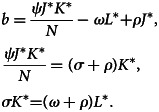


*Theorem 6:* If *R*_0_ > 1, then the model given in (1) is globally asymptotically stable at *E*_1_ when *ω* = 0.

*Proof:* Using the method proposed by [23], we define the subsequent Lyapunov function for equation (1):31
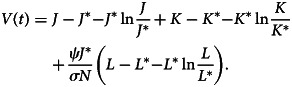


After differentiating equation (31) with respect to time *t*, we have32
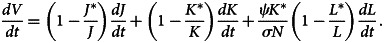


Now,33
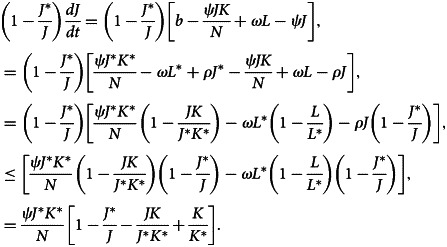
34
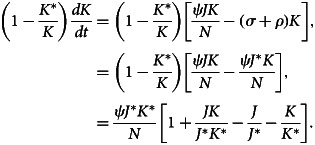
35
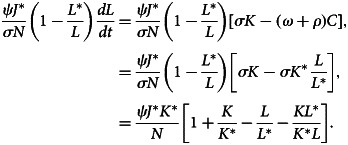
When the outcomes of equations (33)–(35) are substituted to equation (32), we obtain36
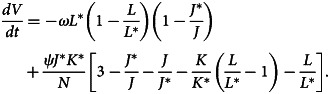


Here, (*dV*/*dt*) ≤ 0 if [3 − (J*/*J*) − (*J*/J*) − (*K*/K*)((*L*/L*) − 1) − (*L*/L*)] ≤ 0. Therefore, using [22], *E*_1_ is globally asymptotically stable whenever *R*_0_ > 1.

### Sensitivity analysis of model parameters

Sensitivity analysis is commonly used for verifying and identifying parameters that can influence the basic reproduction number, *R*_0_, which determines the robustness of model forecasts. It indicates the importance of each parameter for the transmission of disease. According to [24], sensitivity indices authorise us to quantify how much a variable varies when a parameter is changed. When the variable is a differentiable function of parameters, partial derivatives can also be used for constructing the sensitivity index.

*Definition:* The normalised forward sensitivity index of an *R*_0_, which depends differentially on a parameter, *x*_*i*_, is given as:37
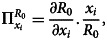
where *x*_*i*_ represents all the basic parameters and *R*_0_ = (*ψ*/*σ* + *ρ*).38
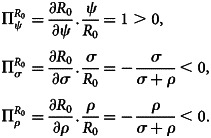


### Interpretation of sensitivity indices

The sensitivity indices of *R*_0_ with respect to the key parameters are shown on Table 2. The positive indices *ψ* show that it has a significant impact on the spread of the disease with increasing values. Since as their values rise, the basic reproduction number increases, and hence the average number of secondary cases of infection rises as well. In addition, those parameters with negative sensitivity indices *σ* and *ρ* have the effect of reducing illness pain when their values increase while the others unchanged. Furthermore, as their values increase, the basic reproduction number decreases, which results in the reduction of the disease's endemic areas.

**Table 2.** Sensitivity indices of the model's parameters

Parameter symbol	Sensitivity indices
*ψ*	+ve
*σ*	−ve
*ρ*	−ve

## Numerical simulation

The numerical results for the system of (1) for various parameter values are presented. The simulation is done by using Matlab ODE45. The values of parameters are given in Table 3. We assumed that the total population and recruitment rate are related by *b* = *ψN*. The initial conditions given below serve as the foundation for the simulations and analyses: *J*(0) = 10 000, *K*(0) = 500 and *L*(0) = 2000.

**Table 3.** Parameter values of the model

Parameter	Value	Source
*b*	*ρ N*	To be computed
*ψ*	0.067	[7]
*σ*	0.007	Assumed
*ω*	0.00021	Assumed
*ρ*	0.012	Assumed

**Fig. 4.**
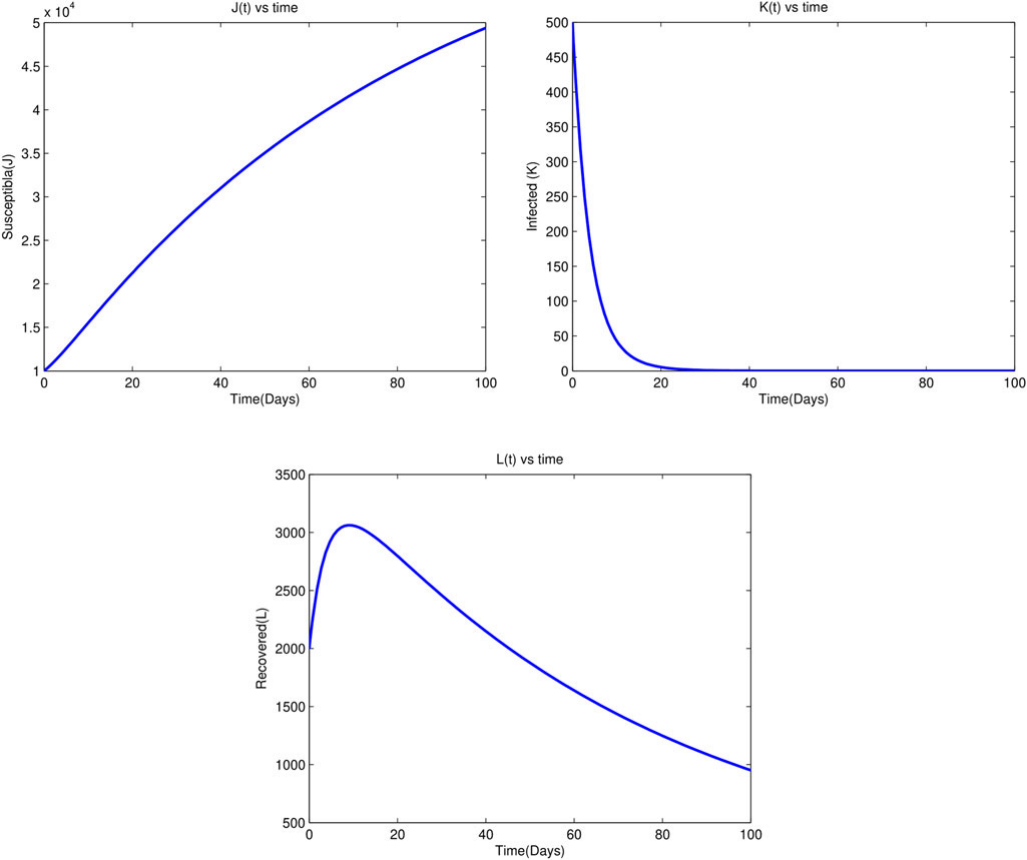
Time series plot of state variables for *R*_0_ = 0.8603 < 1.

From Figure 4, we observe that susceptible and recovered individuals asymptotically increase to the disease-free equilibrium point, while the infected individuals decrease asymptotically to the disease-free equilibrium point. Such conditions exist due to the fact that *R*_0_ = 0.8603 < 1. This supports that the stability of the disease-free equilibrium point exists when *R*_0_ < 1, which means if *R*_0_ < 1, for the duration of the disease period, a single infected individual on average produces less than one newly infected individual.

**Fig. 5.**
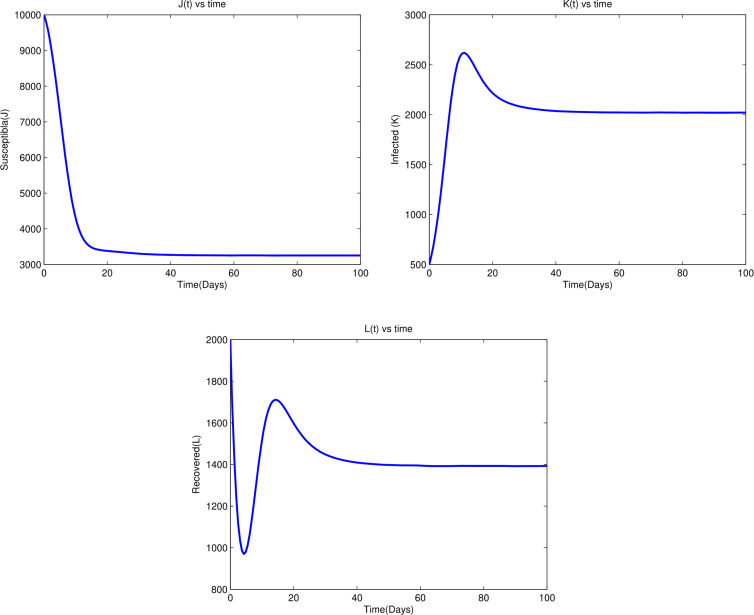
Time series plot of state variables for *R*_0_ = 2.0492 > 1.

From Figure 5, we observe that susceptible and recovered individuals are decreased due to the influence of infected individuals, then they become infected; as a result, the infected individuals are increased. Therefore, infected individuals are increased and the disease endemic equilibrium point exists and stable. The existence of this condition is due to the fact that *R*_0_ = 2.0492 which is greater than one. This supports the theorem that the stability of disease endemic equilibrium point exists when *R*_0_ > 1, this means that when *R*_0_ > 1, on average, each infected person creates more than one other contaminated person, then infected individuals will be able to spread in the given society.

**Fig. 6.**
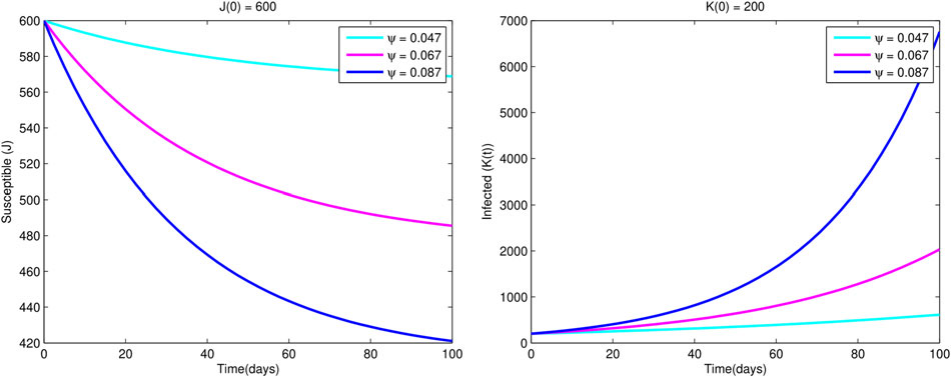
Variations of susceptible population *J*(*t*) and infected population *K*(*t*) w.r.t. time *t* for different values of *ψ*.

Figure 6 shows that as effective contact rate, *ψ* grows, the number of infected people rises while the number of susceptible people falls as a result of the influence of infected people.

**Fig. 7.**
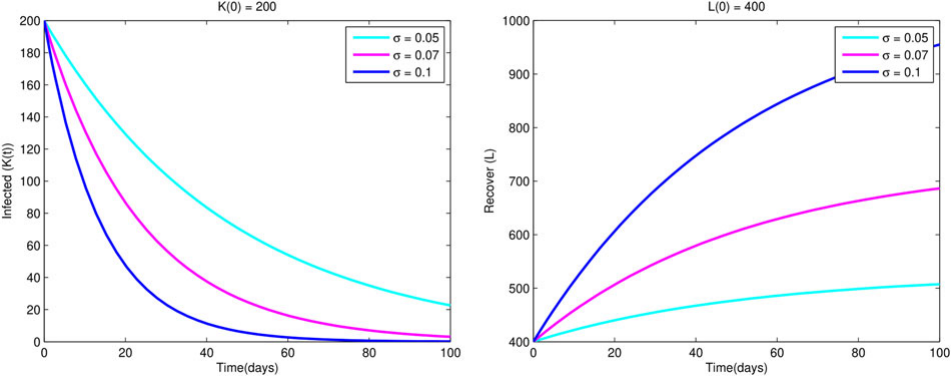
Variations of infected population *K*(*t*) and recovered population *L*(*t*) w.r.t. time *t* for different values of *σ*.

Furthermore from Figure 7, we observe that as the recovery rate *σ* increases, infected individuals decrease while the recovered individuals are increased.

**Fig. 8.**
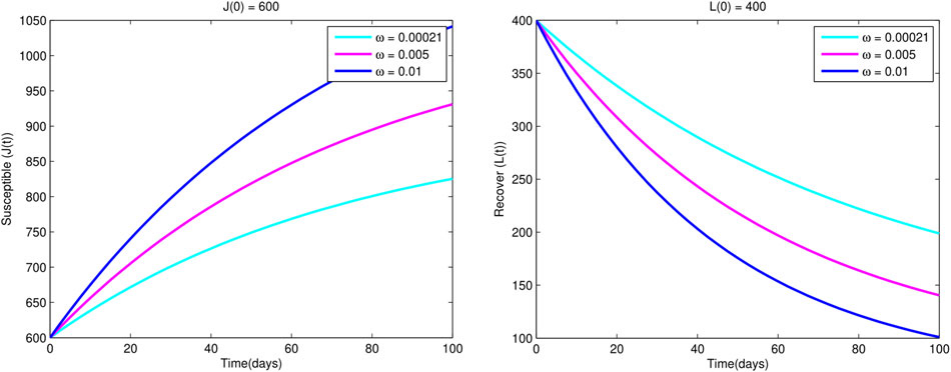
Variations of susceptible population *J*(*t*) and recovered population *K*(*t*) w.r.t. time *t* for different values of *ω*.

Also from Figure 8, we see that as the rate of susceptibility of recovered individuals decreases, recovered individuals are increased while the susceptible individuals are decreased.

## Conclusion

A non-linear deterministic mathematical model for skin sores transmission dynamics was constructed in this work. We first established that the model is well-posed epidemiologically and mathematically. The basic reproduction number (*R*_0_), as well as endemic and the disease-free critical points are determined. The value of (*R*_0_) is used to assess whether equilibrium points are stable or not. When *R*_0_ < 1, the disease-free critical point (*E*_0_) will be both globally and locally asymptotically stable. That is, on average, one infected individual produces less than one newly infected individual over the course of its disease period. As a result, the infected people are gradually removed from the society. Also, the disease endemic critical point (*E*_1_) is globally and locally asymptotically stable if *R*_0_ is greater than 1. That is, each infected individual produces on average more than one new infected individual, then infected individuals will be able to spread in the given society. Consequently, the disease persists in the society. The computational simulations and analyses demonstrate that when the effective contact rate declines and as the recovery rate increases, infected individuals decrease from the society. The model we adapted did not provide optimal control and cost-effectiveness of various intervention strategies that can be explored in the future to find out which strategy is the top in controlling skin sores.

## Data Availability

The data used to support the finding of this study are included in the article. Actually, we used data from other papers for the simulation. The papers are properly cited.
